# Perceived factors informing the pre-acceptability of digital health innovation by aging respiratory patients: a case study from the Republic of Ireland

**DOI:** 10.3389/fpubh.2023.1203937

**Published:** 2023-10-24

**Authors:** Tara Byrne, Niall Murray, Mary McDonnell-Naughton, Neil J. Rowan

**Affiliations:** ^1^Saolta University Healthcare Group, HSE, Galway, Ireland; ^2^Department of Nursing and Healthcare, Technological University of the Shannon (TUS), Athlone, Ireland; ^3^Faculty of Engineering and Informatics, Technological University of the Shannon, (TUS), Athlone, Ireland

**Keywords:** digital health, respiratory health, education, living labs, quintuple Helix hub, aging

## Abstract

It is appreciated that digital health is increasing in interest as an important area for efficiently standardizing and developing health services in Ireland, and worldwide. However, digital health is still considered to be in its infancy and there is a need to understand important factors that will support the development and uniform uptake of these technologies, which embrace their utility and ensure data trustworthiness. This constituted the first study to identify themes believed to be relevant by respiratory care and digital health experts in the Republic of Ireland to help inform future decision-making among respiratory patients that may potentially facilitate engagement with and appropriate use of digital health innovation (DHI). The study explored and identified expert participant perceptions, beliefs, barriers, and cues to action that would inform content and future deployment of living labs in respiratory care for remote patient monitoring of people with respiratory diseases using DHI. The objective of this case study was to generate and evaluate appropriate data sets to inform the selection and future deployment of an ICT-enabling technology that will empower patients to manage their respiratory systems in real-time in a safe effective manner through remote consultation with health service providers. The co-creation of effective DHI for respiratory care will be informed by multi-actor stakeholder participation, such as through a Quintuple Helix Hub framework combining university-industry-government-healthcare-society engagements. Studies, such as this, will help bridge the interface between top-down digital health policies and bottom-up end-user engagements to ensure safe and effective use of health technology. In addition, it will address the need to reach a consensus on appropriate key performance indicators (KPIs) for effective uptake, implementation, standardization, and regulation of DHI.

## 1. Introduction

The impact of chronic disease on healthcare systems internationally is well documented (1–3). Effective and resource-efficient long-term management of multimorbidity is one of the greatest health-related challenges facing patients, health professionals, and society more broadly (3). Respiratory disease represents a diverse range of acute and chronic diseases that are a major cause of morbidity and mortality (4). This situation has been exacerbated by the COVID-19 pandemic (5). Respiratory diseases are responsible for a large proportion of the overall health burden of illness, both in Ireland and globally (6). It is estimated that respiratory disease causes one in five deaths nationally which is 38.2% higher than the EU-28% average. In 2018, a report titled “Respiratory Health of the Nation” found that respiratory disease accounted for 14.3% (*n* = 92,391) of inpatient hospitalizations and 15.8% (*n* = 578,319) of bed days. Comparable figures for cardiovascular disease were 8.2 and 11.3%, and for non-respiratory cancers 4.7 and 8.0% (7).

The delivery of healthcare services has witnessed an accelerated evolution in recent years. Healthcare professionals have had to exercise creativity to meet the changing needs of service users (8). For example, there is a commensurate interest in implementing strategies to support remote patient monitoring and telemedicine to help service users at home and to provide follow-up consultations. Digital health is defined by the WHO as a field of knowledge and practice associated with the development and use of digital technologies to improve health (9) There is evidence to suggest that these programs can improve the quality of care and compliance, reduce the financial burden and ultimately improve patients quality of life (10). Remote healthcare is an evolving concept that is seeing clinicians move toward remote monitoring for service users outside of the hospital setting. Malasinghe et al. (11) propose that there are many advantages to this type of healthcare. These include real-time detection of illnesses, prevention of worsening of illness/ untimely deaths, and reduced hospital admission. Noah et al. (12) reported that remote patient monitoring has many positive outcomes; however, caution must be taken by clinicians using remote patient monitoring and further research is required.

According to the report titled “Health in the 21st Century; Putting Data to Work for Stronger Health Systems” recently published by OECD (13), intelligent use of data and digital technology improves the safety and quality of care provided in healthcare. It also helps address unmet health needs and makes accessing services easier. It supports informed health system stewardship and the development of policies. Effective data collection also assists researchers to develop safer and better treatments, and enables more robust disease prevention and public health, resulting in healthier and more productive populations. The Irish government has faced challenges as to how this country will appropriately address its overwhelmed health service as attested by extensive and lengthy patient waiting lists for elective surgery and consultations. Moreover, there is also a growing concern surrounding future predictions of extreme burden due to the prolonged lifespan of the aging population. The concept of “living labs” in health care has been proposed as a framework to connect governmental, public-sector organizations, industry, higher education institutions, community-based organizations, and clinicians. The aim is to create an environment of creativity that encourages a collaborative approach in the developmental process of a product, service, or system.

Globally, the adoption of digital technologies varies significantly (8). There is evidence to suggest that the adoption of wearable technologies has significantly lagged in comparison to other established technologies such as smartphones and tablets. Cheung et al. (14) noted that when it comes to healthcare, researchers have inadequate knowledge of the adoption intentions of service users. A high proportion of the research conducted has a primary focus on the technical development of the device; therefore, there is an inadequate understanding of the diffusion process. This contrasts with the marketing research conducted for smart technologies, which is primarily focused on consumer adoption, resulting in a much quicker diffusion process. Brenner et al. (8) highlighted the significant gap in evidenced-based published literature across 10 databases on the development of key performance indicators for the development of digital health interventions where only five references were eligible. Key performance indicators play a central role in the evaluation, measurement, and improvement of healthcare quality and service performance. This also intimates a gap in knowledge concerning the service users adoption of technology within healthcare. Lycett et al. (15) suggest the use of psychological theory can enhance the effectiveness of digital interventions and ultimately result in more successful outcomes such as increased consumer adoption. The systematic review concluded with a future recommendation for researchers to further evaluate how the application of theory in the development of digital interventions impacts their overall effectiveness. It is suggested that the use of a psychological framework to gain insight and understanding into consumer adoption will lead to positive engagement with digital health technologies. More recently, future recommendations by Nadal et al. (16) identified that the gap in the current body of knowledge was in the pre-acceptance of technologies. A main thrust of research has focused on understanding people’s perspectives before and after using digital health innovations (DIH) where the initial emphasis has been placed on establishing appropriate multi-actor partnerships with relevant stakeholders including end-users, developing models for evaluation and monitoring, informed by best-published evidence, and the generation of key performance indicators (KPI’s) for measuring the effectiveness and appropriateness of DHI that is currently lacking (8). However, if the main goal is to access the effectiveness of DHI, it cannot be assumed that the service user will engage with the technology long term, or indeed at all. Dundon et al. (17) noted that digital tools for diagnosis and management of respiratory conditions are an important area for research and development; however, the long-term success in this domain will depend on identifying real needs and integrating the often-divergent interests of the various partners in healthcare systems worldwide. Thus, the overarching aim of this novel study is to gain an understanding of the pre-acceptability of respiratory patients to digital health technologies in the Republic of Ireland by interviewing key subject matter experts encompassing respiratory care and digital health.

## 2. Methodology

### 2.1. Research approach including philosophical underpinning

A reflective thematic analysis framework (18) that addresses flexibility within data analysis while maintaining the integrity of the method was used in this study. This method for health research is supported by the literature and deemed “*an interpretive method firmly situated within a qualitative paradigm that would also have broad applicability within a range of qualitative health research designs”* (19). This study used a phenomenological approach to explore the participants’ intentions, perceived thinking, and reactions toward digital health. Subsequently, experiences were captured without any prejudice and participants were provided ample space and time to share their experiences. In line with a phenomenological approach, the phase of the study provides a detailed description of participants’ experiences from analysis through to contextualized findings (20).

### 2.2. Participants

Purposive sampling was used in the study to select the participants which has allowed the researcher to choose appropriate members with selected levels of expertise. Samples were not chosen randomly as not every member of the particular specialty is eligible to partake in this study. Pursuing random sampling also needs significantly more time and information, beyond the capacity of this project which led the researcher to use purposive sampling. Saturation is reached at a point where similar themes were provided as answers to the questions posed (21). However, in this particular study, not all questions that had reached saturation were void, as some were retained to expand themes and help with the discovery of new information. Saturation points were discovered as the transcription process occurred simultaneously during the interview process.

### 2.3. Inclusion and exclusion criteria

Participants in this study were Irish women and men. Each participant was invited to partake in the study, as they will have been identified to subject matter experts who possess particular qualities or skills relevant to the digital health technology field and /or Respiratory disease. Subject matter experts participating in this study encompassed a respiratory physician, psychologist, digital health expert, technological expert, respiratory nurse specialist, health innovation representative, and a government representative.

### 2.4. Ethics statement

In qualitative research, ethics is one essential part that must be considered. Ethical approval is important for all types of research to result in a benefit and to minimize the risk of harm, by protecting participants’ information by informing the participants of everything about the study and their roles as participants, and minimizing the misuse of the information given. It is equivalent to a moral contract when it comes to dealing with humans (22). Ethical applications were first sent to the Technological University of the Shannon Research Ethics Committee, and thereafter the clinical sites. The completed submissions were made on 12^th^ Dec 2021 and were approved *via* email on January 15^th,^ 2022. The researcher carried out data collection (interviews) from March 2022 to May 2022. Ethical approval number C.A.2734.

### 2.5. Data collection

Semi-structured interviews were carried out with the participants. The qualitative phase was a crucial level in which the researcher gained a better understanding of behaviors and knowledge among the targeted population (23). Data collection was conducted in English, as it is the first language spoken in Ireland. Before the interviews commenced, participants were first informed through the information sheet that all the information gained from the interviews would be kept completely confidential. Besides informing participants about the study, the information sheet is a comprehensive reference for the participants to refer to; if anything ever happened to them after the interview session. It is also mentioned in that particular document about confidentiality and how the information will be stored and kept confidential using coding to respect anonymity. Participants were also informed of their rights to withdraw from the study at any stage (Protocol included in Supplementary material).

### 2.6. Study setting

The reasons for choosing a small number of participants for this study are as follows. Firstly, it is valuable to understand peoples experiences within their area of expertise in this topic. This helped the researcher gain valuable insight into diverse areas within the area of digital health technology and indeed technology specific to the area of respiratory diseases. It took at least one to 2 days to explore and draw a conclusion after each conversation before starting a new interview. Also, the time schedule for interviews depended on what free time the participant had, and not all who were invited could or were willing to participate in the interview. Secondly, because the locations were separated geographically, the researcher’s time to interview participants was limited, therefore the option of a virtual interview was offered. Thirdly, there were a small number of participants who had the most valuable experiences and were to deliver the expectations of the researcher purposefully. Interviews were carried out until data saturation was reached. Lastly, it is relevant that the number of interviewed participants met the research objectives and fulfilled the research aim. Data collected and analyzed at this qualitative phase 1 were aimed at developing an instrument for a future quantitative phase II. The data collection was performed primarily through Zoom narrative interviews, using open-ended questions. In the interview sessions, questions were asked according to the interview protocols. Participants responses also generated further questions about the study topic. Each interview was recorded, guided by an interview protocol and guide, and also by the recommendation of the regional ethics committees.

### 2.7. Bracketing

In, bracketing is essential for understanding the phenomenology method. In Braun and Clarke’s phenomenological research method, the application of bracketing is a process to prove the validity and to demonstrate the phenomenological approach through the research process, not only during the data collection but also during data analysis. In this particular study, bracketing began to take place as soon as the interview started. Bracketing is important for the researcher to avoid pre-judgment and assumptions.

### 2.8. Reflection

After utilizing bracketing, the researcher used reflection to help improve her understanding of the outcome and the meaning of the findings of this study (20). This activity involves thorough and deep thought of any factor that might contribute toward respondents’ reactions about the studied topic (24). Reflection is an important activity, especially for social science research, where the relationship with scientific needs was established in exploring thoughts through culture (25). The environment and experiences are real and natural; thus, it is categorized as valuable and rich. It also involves recalling and extracting participants’ details such as: who said that, how, when, where, and why. Through this research, the researcher came to learn and appreciate the art of reflection and practiced this process through the analysis of the project findings for phase 1.

### 2.9. Data analysis

Data was analyzed in agreement with Braun and Clarke’s data analysis framework. The researcher explored the data analysis tools available and decided to adapt Braun and Clarke’s framework. Braun and Clark’s framework is one of the most popular frameworks and is used widely by qualitative researchers to gain reliable results. In this study, the adaption of Braun and Clarke’s phenomenological analysis method is appreciated and translated into the following steps: the interviews were conducted and the researcher practiced bracketing during the particular time to ensure original experiences and thoughts were produced by the participants. The raw data from the audio were then transcribed. Subsequently, the researcher decided to use computer-aided qualitative data analysis to help with coding and theming. Through coding, themes emerged accordingly and supported the aim of this study. Emerging themes were either similar or different from one participant to another. Transcripts were uploaded into NVivo to allow the process. NVivo also helped the researcher to see the statements made by the participants being placed under certain themes. Coding data using NVivo saves the researcher time and also helps to organize complex data. From there, themes were extracted, sub-themes were reorganized and data was organized under the identified gaps. These statements were then gathered under matrices. Finally, themes were organized again and this stage eliminated the redundancy of themes, also, all codes evolved were clustered in a bigger theme. The steps have considered the application of NVivo computer-aided data analysis software to aid the analysis process, especially in theming the transcribed data.

## 3. Findings

### 3.1. Surrounding key themes emerging from semi-structured interviews with subject matter-experts in respiratory care and digital health on beliefs and barriers to uptake of digital health technologies by patients

#### 3.1.1. Utility and patient understanding

Participants commented on the ability of patients to appreciate and use digital technologies for personal management of their respiratory symptoms, for example, Participant 1 believed*” I think there’s a little bit of work to be done first before they are given the device around getting them to understand that they can affect change or they can make something at least improve something even if they have a chronic illness that they have control over exacerbation of symptoms*.”

#### 3.1.2. Digital literacy

Digital literacy was noted as a key consideration to the acceptability of technology. Digital health literacy has been identified within the literature as being a factor that influences the adoption of digital health technology but it also is a significant barrier. Slevin et al. (26) explore this theory within their study, where findings suggest that individuals with previous experience with technology, perceived these skills enhanced their digital literacy abilities, therefore empowering them to engage with digital health technology. In the same study, digital literacy was reported to be a significant adoption barrier to digital health technology.

Participant 6 in this study stated “*The ability of the person to use the device is an important consideration. “I have just seen a 47-year-old lady who does not know how to send an email when I tried to give her contact details of how best to contact somebody in an emergency or if they have a question.”*

#### 3.1.3. Data privacy and trustworthiness

Participants noted that despite concerns, the use of technology can have a relatively positive impact on people’s lives as noted by Participant *5 “Technology has changed our lives, you know technology is a good idea for the most part.”*

Korpershoek et al. (27) suggest that individuals do not feel that digital health technology can be trusted. Data privacy is commonly discussed and an area that internationally raises concern. The Data Protection Acts 1988–2018 are designed to protect people’s privacy. The legislation confers rights on individuals concerning the privacy of their data as well as responsibilities on those persons holding and processing such data. It is assumed that individuals may have strong opinions on their health data and how it may be used; however, to note this is only an assumption and confirmation would be beneficial. Interestingly multiple subject matter experts in this study did not feel that service users would have significant data privacy concerns.

Participant *4* believed that *“I’m not sure about privacy, I do not think that’s as big an issue as it may be, for some people, but not for everyone, I think it’s getting across and understanding what it is in the first instance, and how your data is being used, and when it is your health data for the people who are the controllers and are the ones who are making these decisions for their clinical team, they need this information.”*

Participant 5 stated *“I do not know if the service users in the patient cohort have huge data concerns. I do not feel like you know patients come in and say God that looks amazing but I’m worried that the Russians are looking you know I mean I just do not.”*

Participant 6 stated “*No, I would not have said that in fact, I would consider the consultation, a lot more privacy on digital technology, because there’s a lot of security and protection there for patients with the GDPR concerns some may have, so no I think there’s much more privacy sitting in a room on their own.”*

#### 3.1.4. Equality

Equal and fair access to the necessary amenities to engage in digital health technology is ambiguous. This is a common theme among other studies. Multiple authors such as Mathar et al. (28) and Disler et al. (29) explore the concept that individuals claim that they have no access to technology due to their location and age, but also that they would have little to no confidence in their ability to use any device. It is somewhat unclear what individuals define as “access to.” On one hand, individuals are insinuating that they do not own a piece of technology such as a computer to access some of the available online resources, however, the lack of internet access was also highlighted. None of the studies in the literature made specific reference to the access to internet and the reasons why this was an issue. It is very unclear if the participants in the studies which vary across multiple international countries such as the United Kingdom, Australia, Denmark Norway to name a few, were from an urban or rural geographical location. One study however conducted by Sönnerfors et al. (30) in Sweden, does however mention that data were collected in rural and urban areas however no differentiation was made in the discussion of the results. This study was unique also, as it reported that access to the internet and access to technology was a significant facilitator to the adoption of technology. The author highlights to the reader that in Sweden, approximately only 4% of the population are seldom or non-users of the internet. It is also worth noting that the Swedish government has a national vision of e-health for 2025, in which the government pledges to assist and support the population, to have increased access to the internet and digital devices. This would suggest that the successful adoption of digital health technology would require commitment and support from local government to invest in both rural and urban infrastructure and internet access. It would be safe to assume that rural Ireland would lack similar resources and most definitely requires investment.

Similarly in this study, participants believed that not every individual in Ireland has equal and fair access to digital technologies.

Participant 4″ *“The IT infrastructure might be challenging particularly in rural parts of the country*.”

Participant 6 stated “*high-speed broadband so even though you are kind of maybe saying okay mine maybe that brilliant for the older population, it might be that brilliant for the younger population, because they cannot afford to engage in it.”*

Participant 7 *“Absolutely not and that goes back to you know I’m in a Council House and I am not getting the wifi because they are going to make me commit to 12 months, but the Council said, I have to move out of here in 3 months so I’m not going to sign up there.”*

#### 3.1.5. Education

Education, or more specifically IT education, is mentioned within the literature as both a facilitator and barrier to the adoption of digital health technology. Slevin et al. (26) report from an Irish study that participants perceived that IT education should be personalized for everyone. Personalized early IT education would result in a higher uptake of engagement with digital health technology, as it would instill competence and confidence in individuals when presented with digital health technology.

Participant 6 stated, “*I mean, even in my career like I’m self-learning every single bit of it that I’ve ever done.”* Education regarding the use of technology and the intention that it is in place to support and is not intended to replace the HCP is warranted to negate any ill feeling toward technology.

Participant 1 comments *“I think, as soon as they are put on something that is remote so away from a person and they are feeling like their issues are being trivialized in some way*. *They need to see that it does not have to be an all or nothing, it can be supported by a person and use of technology.”*

Participant 4 believed that “*a lack of protocol on the clinical side and a lack of understanding or awareness of what was happening on the service user side.”*

**Figure 1:**
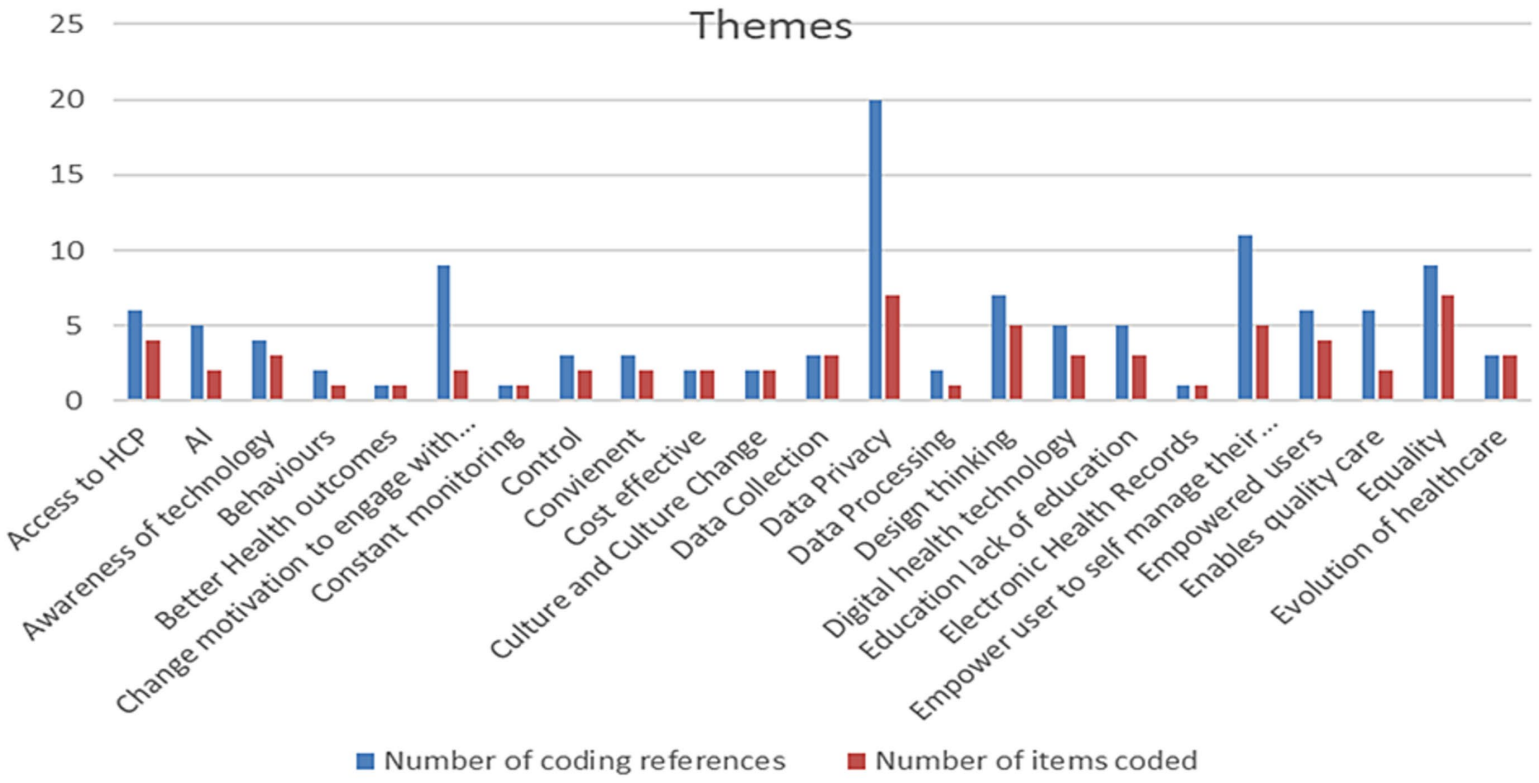
Themes emerging from semi-structured interviews with subject-matter expert participants in this study.

A summary of the themes emerging from the interviews with subject-matter expert participants in this project, on reaching data saturation, is shown in Figure 1.

The data analyzed from these semi-structured interviews highlighted many key themes among the subject matter experts on the role and potential effectiveness of DHI for remote patient use. There was a strong concern that people’s awareness of digital technologies and their perceived usefulness could be poor. There was also concern that poor awareness could hinder the acceptance of technology as people were somewhat blinkered to the advantages. The literature suggests that the increased knowledge and awareness of disease resulted in better self-management, better reported quality of life, and improved continuity of care from healthcare professionals (31). However, not all studies acknowledged this as a facilitator of the adoption to digital health technology. A qualitative study conducted in the United Kingdom by Sanders et al. (32) reflects the perception that engagement with digital health technology poses a threat to an individual’s identity, autonomy, and ability to self-care. It was believed that the use of digital health technology would result in a lifestyle that put too much focus on ill health and would encourage a high degree of dependency on the technology. Individuals were also keen to distance themselves from technology to avoid negative stereotypes of ill health and aging. The increased access to health data and focus in symptom awareness was seen as an aggravating factor for anxiety for some individuals (32) whereas counter-argument was made by Slevin et al. (33) who insinuates that engagement with digital health technology was seen to reduce an individual’s experience of anxiety. It can be concluded that an individual’s perception of usefulness is a significant element that should be considered as a facilitator, but, also a barrier to the adoption of digital health technology. The follow on quantitative phase of this study will encompass translating information from these semi-structured interviews into a questionnaire for respiratory patient participation attending both rural and urban health service clinics. The questionnaire will be developed using data from this study and will apply the Health Information Technology Acceptance Model. This framework is an amalgamation of TAM and HBM (34).

### 3.2. The role of living labs in supporting and enabling development and use of digital health interventions in respiratory care

**Figure 2:**
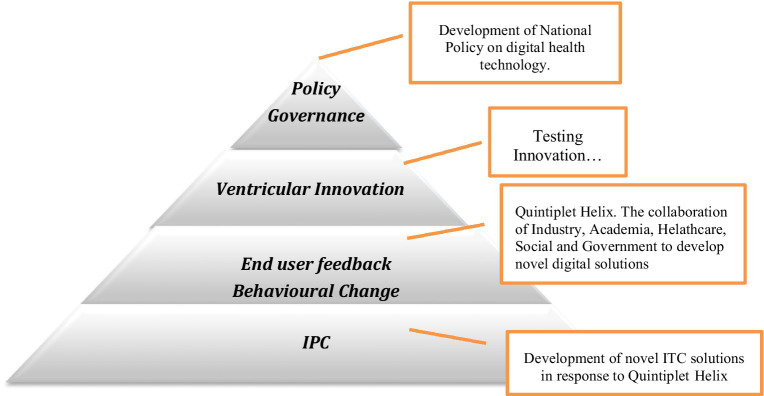
Addressing the interface between top-down digital health policies and service end-user needs, such as through a Quintuple Helix Hub Framework.

Living labs are a relatively new concept within healthcare despite their existence since the early 2000s. There is no commonly accepted definition of living labs, however frequently used adjectives include, open innovation, user-centric, co-creation, test innovation, and real-life context (35). The idea of living labs facilitates the collaboration of knowledge sharing and research design which delivers a user-centered open innovation system. Broadly speaking the key concept of living labs is the idea that a safe space is created to facilitate knowledge exchange, co-ideation, and testing between diverse stakeholder groups in real-life settings (36) The underpinning goal of living labs is to establish and accelerate networking and collaborations of key stakeholders resulting in greater and faster societal impact inclusive of service providers and service users. In Ireland, there are currently nine different living labs focusing on different aspects of digital health. While the type of disease supported varies, the main aim of the living labs is to facilitate the use of technology for remote monitoring, data collection, telehealth, and assistant apps for the older population. Respiratory living labs facilitate actively transferring the research into action. The development of a living laboratory for respiratory care management and intervention in Ireland will be informed by data generated from this study. Key candidate digital technologies to be used and developed in this digital respiratory health library include the Internet of Things (IoT) which includes personalized mobile phone apps; artificial intelligence and machine learning (algorithms) for real-time analysis and intuitive use of big data to promote ease of use and for patient risk mitigation; Edge end-to-end monitoring of data and the use of block chain to develop both business models and to address data trustworthiness; and immersive technologies to help patients and service providers understand new e-technologies. The living lab established for this respiratory patient project or DHI provides access to specialist training environments and subject-matter experts (including immersive technologies), for healthcare and industry through a university interface that also responds to community needs informed by regional policies (Figure 2).

Moreover, the increasing availability and sophistication of mobile health technology continue to garner research interest (37). Liao et al. (37) noted that mobile technology has become a ubiquitous part of everyday life and is challenging the way we offer clinical and health services internationally. However, meeting the challenges posed by unprecedented access to data and the commensurate influx of wearable device data requires a multidisciplinary team of researchers, clinicians, software developers, information technologists, and statisticians. Adoption of digital health technologies in Ireland will also be accelerated by the use of open access and by knowledge transfer from adjacent domains that are more advanced in living laboratories including additive manufacturing and smart agri-food systems (38, 39). The studies of Flott et al. (40) also corroborate the necessity for using this Quintuple Helix Hub encompassing living laboratories as a flexible patient-centered framework for evaluating the digital maturity of health services. Digital maturity is the extent to which digital technologies are used as enablers to deliver a high-quality health service. Flott et al. (40) noted that measurement systems that do exist are limited to evaluating digital programs within one service or care setting, intimating that digital maturity evaluation is not accounting for the needs of patients across their care pathways.

The use of big data and artificial intelligence is under study to stratify the delivery of healthcare. In Ireland, programs have been funded through Horizon (2020), an example being the CLARIFY project which aims to identify risk factors that impact cancer patients’ quality of life after oncological treatment by using Big Data and AI. Data from more than 15,000 survivors of breast, lung, and lymphoma cancer will be reviewed. The objective is to help to stratify cancer survivors by risk to personalize their follow-up by better assessment of their needs.

### 3.3. Quintuple Helix hub framework for support and enabling living labs in respiratory health

The Quintuple Helix Hub framework combines academia-industry-government-healthcare and society thus providing an integrated multi-actor environment enabling digital transformation of living laboratories, such as for bespoke respiratory care and management. There is a pressing need to embrace national digital transformation strategies, particularly for healthcare; however, there is a gap at the interface between top-down strategic policies and bottom-up healthcare and end-users. This framework operates as a one-stop-shop to cross-cut different disciplines that include specialist infrastructure and equipment sharing, subject-matter expertise, demonstrator facilities, human capital building, training and mobility, test-the tech, funding and investing (41, 42). The Quintuple Helix has its’ foundations in previous N-Tuple helices (namely Triple and Quadruple) that are explanatory and active models for facilitating and analyzing knowledge-based economies (43). This author reported that “the Triple Helix model of university-industry-government relations measures the extent to which innovation has become systematic instead of assuming the existence of national (or regional) systems of innovations on a priority ground.” This model also addresses the system of innovation patterns that embraces integrating (such as functions of wealth creation, knowledge production, and normative control taking place at organizational interfaces) and differentiating factors (such as exchanges on the market, scholarly communication in knowledge production, and political discourse) (43). One can determine whether innovation systems are technology-specific or sector-based by review of indicators, such as co-authorship data arising from the Science Citation Index. Leysdesdorff and Sun (44), previously showed that in Japan, “university scholars have increasingly co-authored with foreign colleagues, thus favoring internationalization above relevance when considering the triple helix model of university-industry-government.” It is appreciated that defining selection environments for delineating performing indicators for deploying effective digital health technology beyond the Triple helix of university-industry-government as it will require substantive specification and operational in terms of potentially relevant data that may require the development of additional relevant indicators. However, to effectively deploy appropriate digital technologies, consideration must be given to the additional subject domains of healthcare and society for both subject-matter appreciation, appropriateness, and socio-economic value for tax-payers; thus, inferring development of a Quintuple Helix framework for digital health in Ireland.

This present project addresses key themes for remote patient uptake of digital health innovations including informing future key performance indicators for living labs for respiratory care under a digital health living-lab framework. This challenge is not insignificant, for example, Rowan et al. (39) have noted that there are 706 digital innovation hubs in Europe under varying degrees of maturity. Yet, Brenner et al. (8) highlighted that of the 2,192 publications reviewed and analyzed (PRISMA) between May 2021 and August 2021, only five papers have addressed approaches to inform key performance indicators for the applicability of digital health innovations. Further reading of these five mainly European publications reveals that they mainly focused on developing multi-stakeholder frameworks exploiting literature reviews and expertise meetings to classify indicators (45) and completing interviews with individual stakeholders followed by an interdisciplinary brainstorming session (46). Vedluga (47), applied the Activity Pyramid, Kane’s Model Affinity Diagrams, and Critical quality requirements tree to identify stakeholders, their needs and to determine KPIs for Lithuania’s national eHealth information system. Carrion (48), and Bradway (49), did not include methods to identify KPIs, but described DHI assessment based on principles of technical readiness and maturity, risks, benefits, and resources needed. Thus, there remains a knowledge gap in assessing both the benefits and barriers to supporting and enabling remote respiratory patient monitoring using digital technologies in a Quintuple Helix framework that also addresses appropriate KPIs for reporting on their effective implementation and management, which also embraces feedback to government on policies at the interface with end-users. This present study reports on the first qualitative phase through interviews with subject-matter experts to guide remote aging respiratory patient usage and their empowerment.

Living labs will also be supported and accelerated by digital twin (DT) activities that refer to the “virtual copy or model of any physical entity (physical twin) both of which are interconnected *via* the exchange of data in real-time. Applications of DT include real-time monitoring, designing/planning, optimization, maintenance, remote access, and so forth” (50). Operating an effective living laboratory that exploits digital technologies including digital twin applications for healthcare can increase productivity and efficiency. This Quintuple Helix Hub framework may potentially also operationally meet clinical programs and electronic medical records for the effective commensurate implementation of appropriate technologies into clinical workflow and allow feedback to measure the impact including key performance indicators on clinical outcomes (37). This hub framework can also address the nexus to personalized home healthcare options for smart service delivery and patient-centered monitoring (51), such as respiratory care management. Alexandru and Ianculescu (51), noted that as the number of older adult patients increases with a broad spectrum of needs and specificities, the number of available or caretakers diminishes; thus, the healthcare and social system needs to evolve to meet these trends including informing appropriate and efficient decision making such as financial and human resources.

In the context of specialist training and educational programs delivered in living labs supporting eHealth, Extended Reality technologies such as Virtual reality (VR) are emerging as potential platforms to deliver learning content in a more ecologically valid manner. This is based on their delivery of 360° visuals, spatial audio, and allowing the learner to move beyond the passive mode toward an active participant in their learning experience (52). These technologies in conjunction with various wearable sensor technologies support the capture of various user physiological measures in addition to task performance and user interaction to facilitate a true “human-in-the-loop” system that supports adaptive, personalized while maintaining context-based learning (53, 54). The capture system identifies, at the individual level, key abilities of the learner (by moving beyond binary pass/fail reporting toward understanding a specific individual learning needs). This then informs how the presentation system challenges the learner; thus, optimizing the learner experience. It identifies opportunities for improved training including future provision for operator retraining. The Quadruple Helix Hub framework also supports and enables the integrated knowledge translation (IKT) approach that proposes researcher/knowledge user collaboration as a key step in achieving population impact and a way for society to direct science. IKT shifts from a paradigm where the researcher is an expert to one where researchers and knowledge users are both experts bringing complementary knowledge and skills to the team (55).

### 3.4. Role of DHI as an enabler to informing sustainability for respiratory health

Sustainability is referred to as a societal goal to enable co-existence. More often than not, it is a term more commonly used when referring to global warming and detrimental environmental changes that need radical change. The Irish healthcare system is a constant topic for Government debate which already is at a crisis point. Indeed, with the projected rise in the aging population, the future of Healthcare appears to be grim. The growth of the aging population in the Republic of Ireland has accelerated in comparison to other EU countries. In 2019 the estimated population of individuals greater than 65 in the Republic of Ireland was 696,300 people, which represents approx. 14% of the total population. This is estimated to reach 1.6 million by 2051 (56). This level of growth is likely to increase the already lengthy waiting lists, delay elective surgeries, overburden our emergency departments, and results in poor quality care provision. The current data from January 2023 shows that 505,545 adults and 84,125 children are currently waiting time for Outpatient appointments in Ireland (57). The number of patients waiting for a Respiratory Consultant appointment is estimated to be 19,200. To put this into context, currently, One in eight of the Irish population is waiting for medical intervention. This is 12.5% of the Irish population. Healthcare is at the core of the success of sustainability in many other areas as it is the main beneficiary and contributor to development. It is suggested that ultimately health is determined by a range of environmental, social, and economic influences, and the health of people, places, and the planet are interdependent (58). However, for healthcare to contemplate sustainability, changes need to be radial and imminent.

The introduction and inclusion of technology in the form of digital solutions into how healthcare is delivered is an exciting and welcome innovation currently being explored internationally. Digital solution goals have such diversity, therefore requiring the inclusion of stakeholders who have a particular interest in digital solutions interests (59). Collaboration is the key to success, such as through the Quadruple Helix Hub framework.

### 3.5. Summary

This study aimed to explore the perspectives of subject matter experts and their view of the factors that influence the pre-acceptability of digital health technology in the aging respiratory patient. The common themes identified in the literature were digital literacy, perceived usefulness, education, and access to and reliability of technology. Each theme uniquely impacts an individual’s compliance with digital health technology. Participants discussed the difficulties that they experienced in gaining access to technology and also the lack of availability to the Internet. Most studies in best-published literature did not explore this theme in detail; therefore, it is unclear the reasons for this difficulty. Is it age? Is it geographical? Each of the subject matter experts raised awareness that the availability of appropriate infrastructure was a concern and that not all service users would have access to the internet or technological devices. Lack of digital literacy skills, IT education, and/or access to technology were also identified as concerns that may lead to poor engagement by service users. This topic is somewhat under-researched, and there are very limited Irish studies available for review. Data privacy was also a common theme among the participants in this study, but not a concerning one. It was suggested that service users may be very forthcoming about sharing their health data for the purposes of obtaining support and guidance from healthcare professionals and ultimately disease control. Healthcare is significantly evolving into the world of digital health technology; however, it is very unlikely that service users are evolving as rapidly to evoke change; understanding is needed of the perspectives of the service users to encourage engagement with digital health technology. It is imperative to ensure not only the success of digital health technology but also the sustainability of the Irish healthcare system so that the service users are identified as key stakeholders. Investment in digital health technology is futile if it is not accepted by the end user. Given the increasing emergence of digital innovation hubs across Ireland and Europe (*n* = 206), applying an effective Quintuple Helix Hub framework that encompasses living lab activities will help define datasets and domains for improved utility and data trustworthiness.

This constituted the first study to identify themes believed to be relevant by respiratory care and digital health experts in Ireland to help inform future decision-making among a cohort of respiratory patients in the Irish midlands and Western region that may potentially facilitate engagement with an appropriate use of digital health technology. The study explored and identified expert participant perceptions, beliefs, barriers, and cues to action that would inform content and future deployment of living labs in respiratory care and related strategies for remote patient monitoring of people with respiratory diseases. The ultimate goal of this case study was to generate and evaluate appropriate data sets to inform the selection and future deployment of an ICT-enabling technology that will empower patients to manage their respiratory systems in real-time in a safe effective manner through remote consultation with health service providers. Findings will advance Digital Health Strategies in Ireland and Europe and will have a global orientation. This study focused on respiratory patients only as it is the area of expertise of the researcher in nursing. The researcher is working full-time as an Advanced Nurse Practitioner and is undertaking this study independently. Leave has not been permitted to expand this study; therefore, this novel study focuses on the group of participants that are accessible.

## Data availability statement

The raw data supporting the conclusions of this article will be made available by the authors, without undue reservation.

## Ethics statement

The studies involving humans were approved by Technical University of the Shannon Saolta Hospital Group ethics committee. The studies were conducted in accordance with the local legislation and institutional requirements. The participants provided their written informed consent to participate in this study.

## Author contributions

TB, NR, NM, and MM-N contributed to the ideation, methods development, and review of first draft, proof editing and final review. NR and TB generated first draft of paper. TB obtained funding via PhD project. NR, NM, and MM-N supervised PhD for data generation and paper synthesis. All authors contributed to the article and approved the submitted version.
