# Supplementary material for: Perceived factors informing the pre-acceptability of digital health innovation by aging respiratory patients: a case study from the Republic of Ireland
Source: Front Public Health. 2023 Oct 24;11:1203937. doi: 10.3389/fpubh.2023.1203937 (PMC10628059; doi:10.3389/fpubh.2023.1203937)
Supplement: Supplementary file 1 [file Data_Sheet_1.PDF]

**Key Aim of Research:**

Identification of the perceived factors that influence the acceptability of digital health technology in the aging respiratory patient.

**Researcher**: Tara Byrne, PhD Candidate TUS (Technological University of the Shannon), RANP Respiratory, Saolta Hospital Group.

**Knowledge and Awareness**

1. What is your understanding of digital health technology?
2. How did you come to learn about digital health technology?
3. Can you tell me about some of the remote patient monitoring devices that you currently use or aware of in use?
4. Can you tell me about some of the digital health technology devices that are currently available?

**Perceived ease of use/ Usefulness/ acceptance**

1. Have you ever been approached to be involved in the design process of a digital health device?
2. Do you think if service users/you were involved in the design process, that it would encourage them/you to use technology either personally or recommend it to service users?
3. Do you think there would be greater acceptability of these technologies if service users were involved in the design process of these devices?

**Perceived susceptibility**

1. What in your opinion might motivate a change in a person's behaviour that results in increased susceptibility to digital health technology?

**Perceived Benefits**

1. What in your opinion are the benefits to using digital health technologies?
2. Do you feel that the use of digital health technology would have a positive impact on the health of service users? If yes, why?

**Perceived Barriers**

1. What, in your opinion are the barriers to the success of digital health technologies? Please explain your answer.
2. In your opinion, are people's fears of privacy a barrier to digital health technology?
3. Do you think that a lack of IT/Digital literacy contributes as a barrier to digital health technology?
4. How do you think that issue could be addressed nationally?

**Health motivation**

1. Do you think that the use of digital health technology would empower you/service users to self-manage their disease?
2. In your opinion would the use of digital health technology encourage users to be more self-aware to changes in the symptoms of their respiratory disease?
3. Compliance within healthcare is a common issue. In your opinion what would encourage service users to continue engaging with digital health technology long term?

**Additional Questions**

1. Is there anything that you would like to suggest that might improve digital health technologies for Respiratory Patients?
2. Do you feel that respiratory patients throughout Ireland have equal and fair access to digital health technologies that are currently available?
3. In your opinion does the availability of digital health technology vary depending on the institution/hospital that you attend?

**Closing**

1. Are there any other comments that you would like to add?

**Thank you for participating in this interview.**
